# Poly(vinyl alcohol)/Chitosan/Glycine Composite-Based Eco-Friendly Biodegradable Triboelectric Nanogenerators

**DOI:** 10.1021/acsomega.5c12532

**Published:** 2026-05-16

**Authors:** Tulsi Paudel, Cheng-Tang Pan, Cheng-Yi Chen, Yow-Ling Shiue, Muhammad Sadiq Rahim

**Affiliations:** † Department of Mechanical and Electro-Mechanical Engineering, 34874 National Sun Yat-sen University, Kaohsiung 804201, Taiwan (R.O.C.); ‡ National Center for Instrumentation Research, National Institutes of Applied Research, Hsinchu City 300092, Taiwan (R.O.C.); § Institute of Advanced Semiconductor Packaging and Testing, College of Semiconductor and Advanced Technology Research, 34874 National Sun Yat-sen University, Kaohsiung 804201, Taiwan (R.O.C.); ∥ Institute of Precision Medicine, 34874 National Sun Yat-sen University, Kaohsiung 804201, Taiwan (R.O.C.); ⊥ Department of Electrical Engineering, 63330 Cheng Shiu University, Kaohsiung 833301, Taiwan (R.O.C.); # Institute of Biomedical Sciences, 34874 National Sun Yat-sen University, Kaohsiung 804201, Taiwan (R.O.C.)

## Abstract

Triboelectric nanogenerators (TENGs) offer a low-cost approach for harvesting ambient mechanical energy; however, most devices rely on synthetic, nonbiodegradable polymers. This study presents an eco-friendly biodegradable TENG constructed using a ternary composite of poly­(vinyl alcohol) (PVA), chitosan (CS), and glycine (GL). Moderately transparent PVA/CS/GL composite films were fabricated via a simple in situ solvent-casting process using physical mixing and ambient drying, avoiding toxic solvents and complex thermal processing. The composite film served as the tribo-positive layer, while a polydimethylsiloxane film served as the tribo-negative layer in a vertical contact–separation configuration. The GL content (0–25 wt %) was varied to evaluate its effect on the film structure, mechanical properties, and triboelectric performance. SEM showed GL-dependent surface texturing, and FTIR confirmed the presence of polar functional groups (−OH, −NH_2_/–NH_3_^+^, and −COO^–^) and interaction changes consistent with hydrogen bonding within the composite network. GL incorporation also improved flexibility and mechanical stability compared with the pristine PVA/CS film. The GL20 film exhibited the best electrical performance, delivering an open-circuit voltage of ∼70 V at 10 Hz and a short-circuit current of ∼9–10 μA at a low external resistance, with a peak current of ∼4 μA near the matched load. A maximum power density of 12–13 μW cm^–2^ was achieved at an external load resistance of 5.1 MΩ. The rectified output powered LEDs and charged capacitors (0.22–10 μF), with a 0.22 μF capacitor reaching ∼2.3 V within 40 s. The EB-TENG maintained a stable output over 7500 operating cycles and showed rapid hydrolytic mass loss in phosphate-buffered saline within 1 week. Overall, these results demonstrate the potential of PVA/CS/GL-based EB-TENGs as sustainable and biodegradable power sources for eco-friendly self-powered electronics.

## Graphical abstract

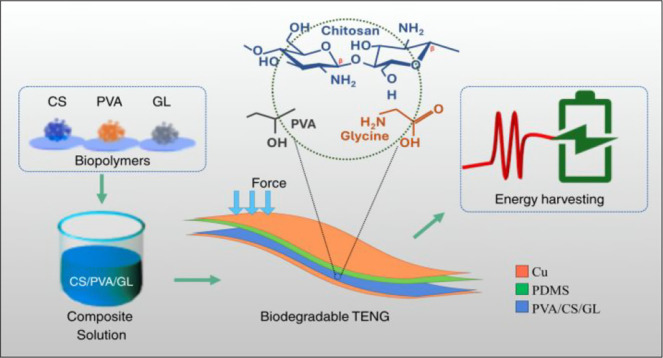

## 1 Introduction

The growing demand for self-powered sensors and environmentally friendly energy-harvesting devices has opened the door to triboelectric nanogenerators (TENGs). TENG devices are versatile and cost-effective for converting mechanical energy into electrical energy via contact electrification and electrostatic induction.^1,2^ Their lightweight and flexible architecture as well as their compatibility with diverse materials make them highly attractive for wearable sensors, biomedical devices, and environmental monitoring.^3,4^ Various materials have been used to produce TENG devices depending on the application and environment.^5^ Despite significant advancements, most high-performance TENGs rely on nonbiodegradable materials that are difficult to dispose of in an environmentally friendly manner. Furthermore, the persistence of these materials in the environment contributes to pollution and waste accumulation owing to their nonbiodegradable nature.^6^ Consequently, there is increasing interest in biodegradable, biocompatible, and low-toxicity components for low-power electronics and health-monitoring applications.^7^ However, TENGs constructed with degradable materials are limited despite the abundance of such biopolymers. Recent reviews highlight rapid growth in biodegradable/biobased TENGs for transient and wearable electronics but also emphasize the need to balance output, mechanical durability, and degradation behavior.^8,9^

In addition to their abundance, many biomaterials are attractive for TENGs because they are renewable and biocompatible and can be processed into flexible films. For example, chitosan (CS), a natural polysaccharide derived from chitin, is known for its biodegradability, biocompatibility, and ability to form flexible films.^6,10^ The amino and hydroxyl functional groups in CS participate in hydrogen bonding and surface charge transfer during triboelectrification, thereby enhancing the charge generation.^11^ Its antimicrobial activity, nontoxicity, and increased utilization in TENG devices, particularly in wearable health electronics, have been reported.^7,11,12^ In this study, we integrated CS with a synthetic polymer, poly­(vinyl alcohol) (PVA), which is water-soluble, nontoxic, and exhibits excellent film-forming properties with extended biodegradability.^10^ The incorporation of PVA improves the flexibility, stability, and mechanical robustness of CS-based films.^13^ Glycine (GL) is another biomaterial that is rarely used in energy harvesting and self-powered applications. It is the simplest amino acid and exhibits polymorphism with distinct crystalline phases (α, β, and γ). α-GL has been reported to possess an antiparallel layered hydrogen bond network and to be the thermodynamically stable polymorph at room temperature.^14^ It is centrosymmetric, whereas β- and γ-GL are noncentrosymmetric and have been associated with piezoelectric behavior.^15–17^ Recently, GL- and amino-acid-enabled nanogenerators have attracted renewed attention because amino acid functional groups can modulate interfacial polarization and charge trapping, and surface potential-based analyses have been increasingly used to correlate molecular chemistry with triboelectric output.^16^

Although CS, PVA, and GL have been individually studied for various energy and biomedical applications, their integration into a single triboelectric composite film remains unexplored. For instance, Liu et al.^18^ fabricated a PVA/γ-GL sandwich-like heterostructured hybrid piezo-TENG that achieved an output of 94 V and 2.3 μA with a current density of 1.53 μA cm^–2^ using PLGA as the tribo-negative layer. Similarly, Ukashi et al.^17^ employed γ-GL with CS to construct a high-performance TENG (*V*_oc_ = 79 V, *I*_sc_ = 64 μA, and *P* = 705 μW at 1 MΩ). These studies primarily explored γ-GL because its noncentrosymmetric structure can exhibit stronger piezoelectric characteristics.^19^ In particular, the role of GL in triboelectric mechanisms has rarely been investigated, and only a limited number of recent studies have attempted to quantify amino acid-induced interfacial charge behavior using surface potential/charge-mapping approaches.^19,20^ Only a limited number of TENG studies have incorporated GL into two-component formulations, and no published reports have described ternary PVA/CS/GL composites. Moreover, because α-GL is centrosymmetric (and thus not expected to provide a strong intrinsic piezoelectric contribution), it has been selected less frequently for energy-harvesting device development.

In this study, we fabricated an eco-friendly biodegradable triboelectric nanogenerator (EB-TENG) using PVA, CS, and GL via a simple solvent-casting method under ambient conditions. We used commercially available α-GL, which has been less extensively investigated than the β- and γ-forms, because it is widely reported as the stable polymorph under ambient conditions and is readily accessible for reproducible film fabrication.^21,22^ This choice also aligns with recent discussions emphasizing processability and reproducibility of amino acid-containing films for scalable energy-harvesting platforms beyond purely maximizing piezoelectric contribution.^8,9,17^ In addition, incorporation of GL into polymer matrices has been reported to influence polarization-related interactions and interfacial charge behavior, which may contribute to improved charge stability in composite devices.^18^ Furthermore, we systematically examined how GL incorporation affects the structural, surface, and electrical properties of the composite film. The results highlight the potential of ternary PVA/CS/GL interactions for next-generation biodegradable sensors, wearable electronics, and green-energy systems.

## 2 Materials and Methods

### 2.1 Materials

Natural CS (medium molecular weight; degree of deacetylation ≥75%) and PVA (average molecular weight ≈89,000–98,000 g mol^–1^; 99+% hydrolyzed) were obtained from Sigma-Aldrich (St. Louis, MO, USA). GL (≥99% purity; commercially available α-GL) was purchased from Uniregion Biotech Co., Ltd. (Taiwan) and used as received. Polydimethylsiloxane (PDMS; Sylgard 184 silicone elastomer base and curing agent) was purchased from Dow Corning Corporation (Midland, MI, USA). Copper (Cu) electrodes, adhesive tape, and deionized (DI) water were obtained from the local market.

### 2.2 Sample Preparation and Fabrication

As shown in Figure 1, the overall sample preparation process involves solution preparation, mixing, casting, and drying. First, 0.43 g of CS was dissolved in 4.33 g of acetic acid and 7.22 g of DI water and stirred at 50 °C and 300 rpm for 3 h until a clear, viscous solution was obtained. In parallel, 0.87 g of PVA was completely dissolved in 2.71 g of DI water at 60 °C under stirring (300 rpm) for 3 h to obtain a transparent solution. The two polymer solutions were then blended at a 2:1 (PVA:CS) weight ratio to form a homogeneous base matrix. Predetermined amounts of GL were added to the PVA/CS mixture to obtain composite solutions containing 5, 10, 15, 20, and 25 wt % GL. The final mixtures were magnetically stirred at 300 rpm for 3 h to ensure uniform dispersion. The resulting smooth, bubble-free solutions were cast into clean rectangular molds (4 × 3 cm^2^) and dried at room temperature for 24 h to produce flexible free-standing films (Figure S1). After drying, the films were carefully peeled off and stored in a desiccator prior to device assembly. Film thickness was measured using a digital micrometer at five locations (center and four corners). Thickness and within-film uniformity (mean ± SD, *n* = 5 points; CV%) are reported in the Supporting Information (Table S1). For the films used in device testing, the thickness was typically ∼0.04–0.07 mm based on the five-point measurements.

**Figure 1:**
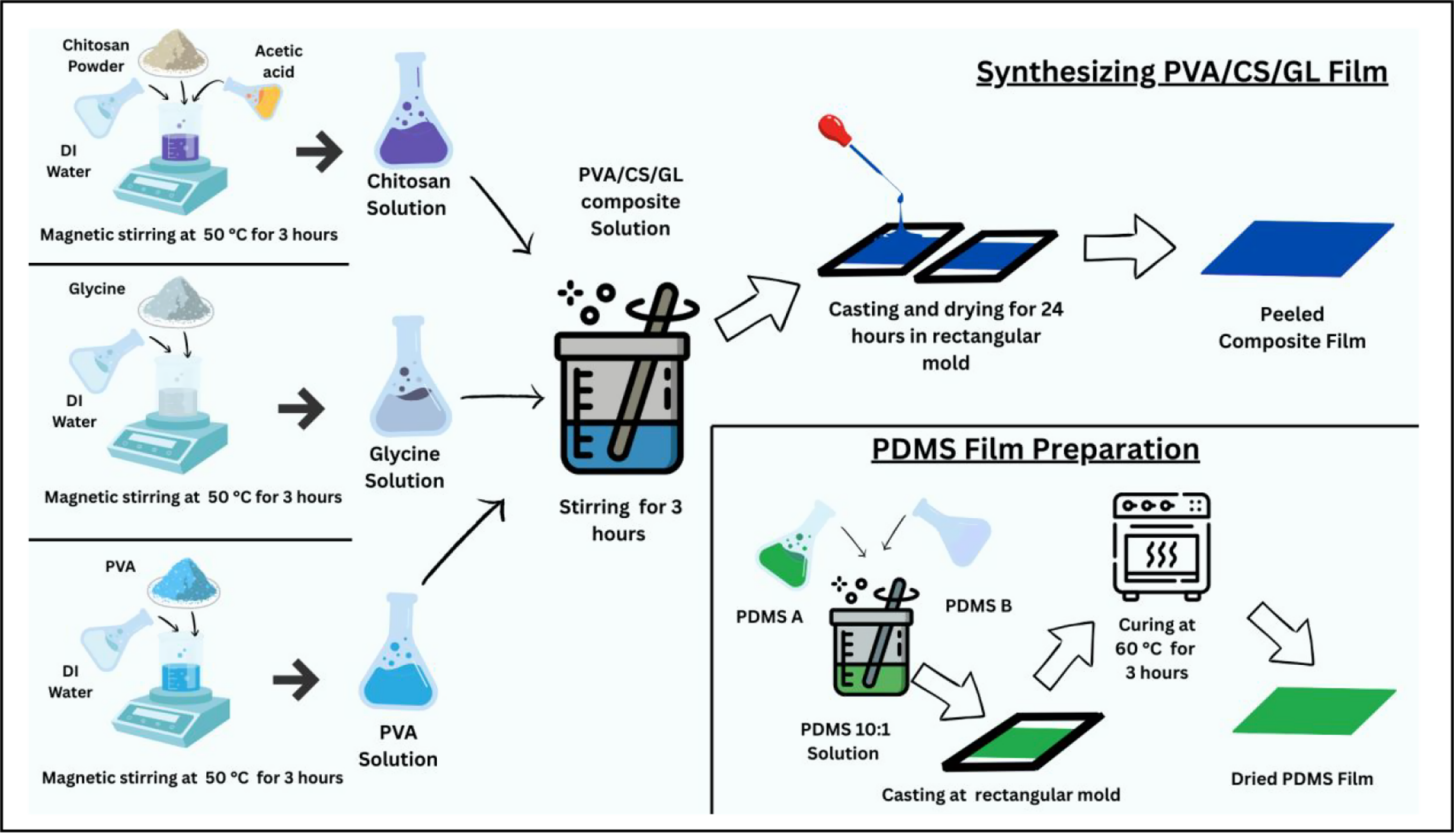
Preparation steps for the PVA/CS/GL composite film and PDMS film, including individual solution formation, mixing, solvent casting, and curing procedures (attribution: iIllustration was created using Canva software).

The PVA/CS/GL composite films were cut into 3.0 × 2.0 cm^2^ pieces and used as the tribo-positive layer of the EB-TENG. For the tribo-negative layer, a PDMS film was prepared by mixing the prepolymer and curing agent at a 10:1 ratio and curing at 60 °C for 3 h. Conductive copper foil was attached to the back of each film as the electrode. The composite biofilm and the PDMS film were assembled in a vertical contact–separation configuration with a 3 mm gap, enabling periodic contact and release under mechanical excitation. The effective contact area during testing was *A* = 6 cm^2^. Device assembly was performed under ambient laboratory conditions, and the devices were tested after a brief stabilization period.

### 2.3 Material and Electrical Characterizations

The structural, morphological, and mechanical characteristics of the composite biofilm were analyzed to correlate its physical properties with its triboelectric behavior. The surface morphology was examined using a field emission scanning electron microscope (ZEISS GeminiSEM 450) operated at 3 kV after sputter coating with a ∼5 nm gold layer to prevent sample charging. Fourier transform infrared (FTIR, PerkinElmer Spectrum 2) spectroscopy was conducted in the 4000–500 cm^–1^ range using the attenuated total reflectance mode to identify functional groups and interaction changes within the composite. Tensile tests were performed using a SHIMADZU EZ-LX (500–5000 N) at a strain rate of 20 mm·min^–1^ at room temperature under ambient laboratory conditions (*T* = 25 ± 2 °C, RH = 70 ± 10%).

Electrical performance tests were conducted using a mechanical shaker (Dataphysics Signal Force GW20) at frequencies of 3–10 Hz with an Agilent function generator at a contact force of 10 N. The electrical output of the fabricated EB-TENG was measured using an oscilloscope (GW Instek GDS-210A) during vertical contact–separation between the composite biofilm and PDMS. The open-circuit voltage (*V*_oc_) and short-circuit current (*I*_sc_) were recorded, and the output power density (PD) was calculated across external load resistances (10 kΩ–15 MΩ) using

$$\mathrm{PD}=\frac{V^{2}}{R\times A}$$

where *A* is the effective contact area, *V* is the output voltage, and *R* is the load resistance.

Electrical measurements were performed under ambient laboratory conditions (*T* = 25 ± 2 °C, RH = 70%). To evaluate device-to-device reproducibility, the optimized formulation (GL20) was characterized using *n* = 3 independently fabricated devices, and the results are reported as mean ± standard deviation (Supporting Information, Table S3).

## 3 Results and Discussion

### 3.1 Physical Appearance and Surface Morphology

A transparent, flexible PVA/CS/GL film that can be bent and stretched is shown in Figure 2a. This macroscopic appearance indicates uniform film formation and suitability for triboelectric applications. SEM analysis revealed pronounced GL-dependent differences in surface morphology. The pristine PVA/CS film (GL0; Figure 2b) exhibited a smooth, dense, and uniform surface without obvious pores or cracks, indicating good compatibility between PVA and CS and providing baseline morphology prior to GL addition. In contrast, the GL-containing PVA/CS/GL film (GL20; Figure 2c) exhibited a more textured surface with granular microdomains, suggesting microstructural reorganization and localized GL-associated regions. Across the full series (GL0–GL25), the high-magnification SEM images (Figure S2) show a GL-dependent evolution of surface microtexture, with granular/crystallite-like domains becoming more apparent at intermediate loadings. Such microtexture evolution is expected to increase the effective contact area and provide additional sites for charge trapping/retention at the triboelectric interface.^18^

**Figure 2:**
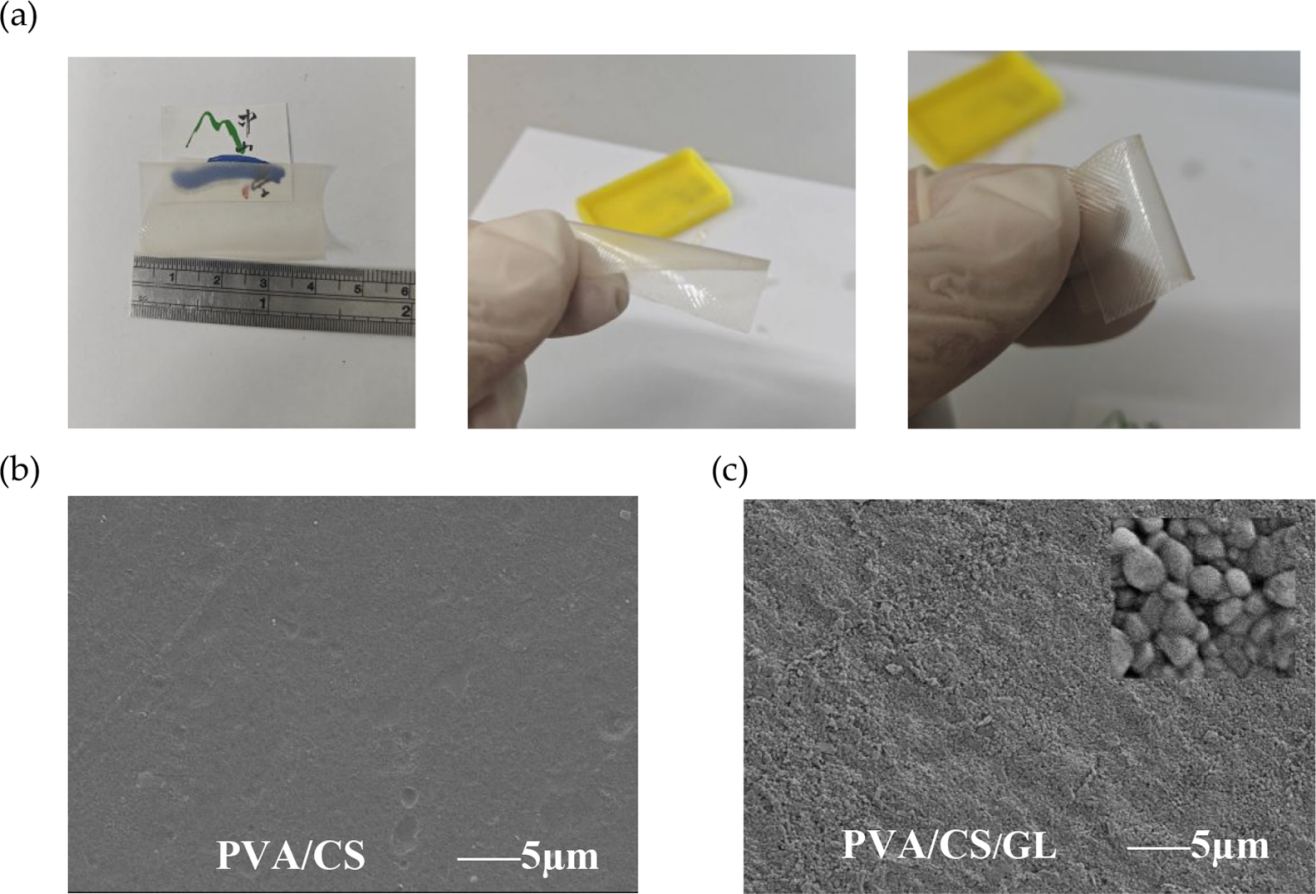
(a) Photographs of flexible PVA/CS and PVA/CS/GL composite films. (b) SEM image of the pristine PVA/CS film. (c) SEM image of the PVA/CS/GL film with a magnified inset showing granular microdomains.

EDS analysis further supports GL incorporation into the composite matrix. The baseline PVA/CS film without GL (GL0) showed no detectable nitrogen signal (*N* = 0.00 wt % and 0.00 at%; Figure S3a and Table S2), whereas the GL-containing film (GL20) exhibited a clear nitrogen signal with an EDS-derived composition of C: 38.43 wt %, N: 16.18 wt %, and O: 45.39 wt % (Figure S3b and Table S2). This contrast provides quantitative evidence that nitrogen-containing species are introduced upon GL addition. Consistent with the SEM-observed microtexture changes, GL incorporation is expected to increase the density of polar functional groups and modulate interfacial charge trapping/retention, contributing to the enhanced electrical output observed for GL-containing films. Quantitative roughness metrology (Ra, Rq, Rz) and spatially resolved surface elemental mapping (e.g., EDS line scans or XPS) across all GL loadings will be pursued in the future work to further strengthen the structure–performance correlation.

### 3.2 FTIR Spectroscopic Analysis

FTIR analysis was performed to identify the characteristic functional groups and assess the interaction changes among PVA, CS, and GL (Figure 3). PVA exhibited a broad band centered at ∼3400 cm^–1^ corresponding to O–H stretching vibrations, together with characteristic peaks at 2920 cm^–1^ (C–H stretching) and 1090 cm^–1^ (C–O–C stretching). CS displayed absorption bands at ∼3200–3500 cm^–1^ (overlapping O–H and N–H stretching), 2875 cm^–1^ (C–H stretching), 1660 cm^–1^ (amide I, C=O stretching), and 1550 cm^–1^ (amide II, N–H bending), which are typical of partially deacetylated CS. GL showed well-defined bands at 1615 cm^–1^ (νas­(COO^–^)), 1510 cm^–1^ (δ­(NH_3_^+^)), 1410 cm^–1^ (νs­(COO^–^)), and 890 cm^–1^ (NH_3_^+^ rocking), which are consistent with literature-reported GL signatures.^16^

**Figure 3:**
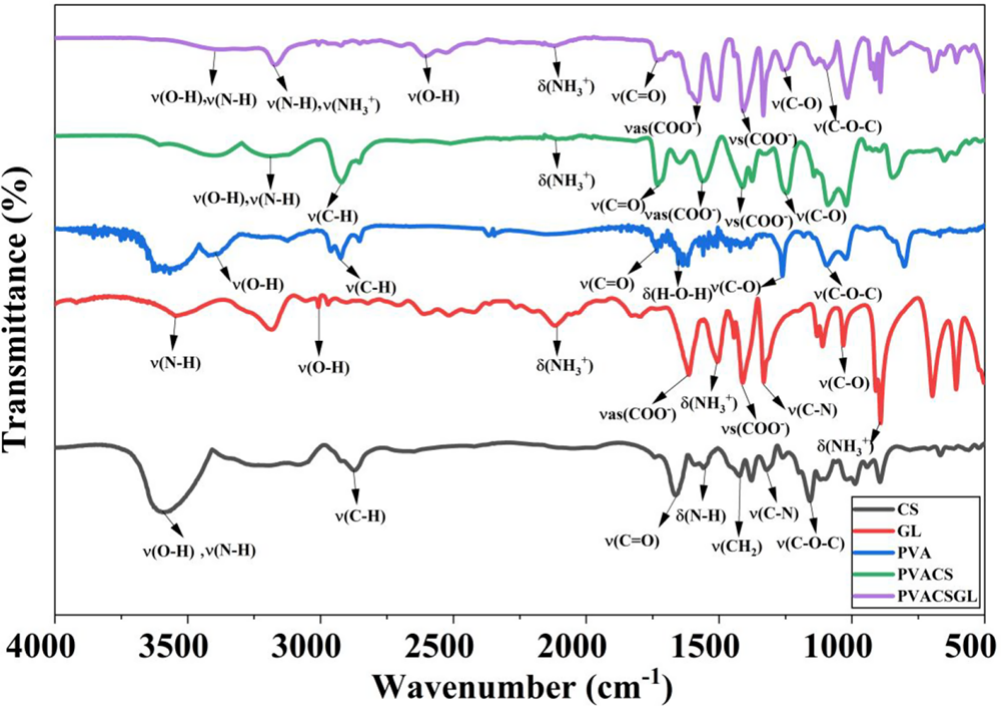
FTIR spectra of pure CS, GL, PVA, PVA/CS, and PVA/CS/GL composite films. The characteristic absorption bands correspond to O–H and N–H stretching (∼3300 cm^–1^), C–H stretching (∼2910 cm^–1^), amide I (C=O, ∼1635 cm^–1^), amide II (N–H bending, ∼1561 cm^–1^), COO^–^ asymmetric and symmetric stretching (∼1600 and 1410 cm^–1^), and C–O–C stretching (∼1070 cm^–1^).

For the binary PVA/CS composite, the spectra revealed broadening of the O–H/N–H region (3200–3500 cm^–1^) and a slight shift of the amide I band from ∼1650 to ∼1643 cm^–1^, indicating hydrogen bond formation between the hydroxyl groups of PVA and the amino groups of CS.^10^ Compared with the baseline PVA/CS film, the ternary PVA/CS/GL composite (optimized GL20 formulation) exhibited additional changes in the O–H/N–H stretching envelope and the appearance/intensification of GL-related bands near ∼1615, 1410, and 890 cm^–1^. These spectral features support the incorporation of GL and suggest strengthened intermolecular interactions within the composite, including hydrogen bonding and electrostatic interactions associated with NH_3_^+^/–COO^–^ functional groups. The decreased intensity near ∼1080 cm^–1^ (C–O–C stretching) is also consistent with the redistribution of intermolecular interactions and local chain environments upon GL incorporation rather than covalent cross-linking.

From a triboelectric perspective, such interaction changes are relevant because polar functional groups (−OH, −NH_2_/–NH_3_^+^, and −COO^–^) can contribute to interfacial polarization and charge trapping/retention during repeated contact–separation cycles.^17^ Therefore, GL incorporation is expected to modulate the interfacial charge behavior of the composite film, which is consistent with the observed enhancement in electrical output.^16^ ^,^ ^23^

It is noted that the present FTIR comparison focuses on the baseline (GL0) and optimized (GL20) formulations and is intended primarily for compositional confirmation and qualitative interaction assessment. A systematic loading-dependent FTIR analysis across GL0–GL25, including integrated peak area ratio tracking and deconvolution of the 3200–3500 cm^–1^ O–H/N–H envelope, will be conducted in the future work to quantitatively map hydrogen-bonding evolution with GL content. Complementary surface/charge characterization (e.g., surface potential mapping) will also be pursued to better correlate molecular interactions with triboelectric output.

### 3.3 Mechanical Properties Analysis

The mechanical behavior of the composite films was investigated using tensile stress–strain analysis to evaluate the influence of GL on the strength, ductility, and deformation characteristics of the films (Figure S4). The pristine PVA/CS film (GL0) exhibited a high tensile strength exceeding 42–45 MPa but a very low strain-to-failure (<3%), indicating a stiff and brittle nature dominated by strong intermolecular hydrogen bonding between the PVA and CS chains (Figure 4). The mechanical response underwent significant changes upon the incorporation of GL. At low GL contents (5–10 wt %), the films exhibited a noticeable reduction in tensile strength accompanied by a substantial increase in elongation. Specifically, GL5 and GL10 showed tensile strengths of ∼15–25 MPa while achieving markedly higher strain levels of ∼10% (GL5) and >35% (GL10), demonstrating the onset of plasticizing behavior as GL disrupted the dense polymer packing and enhanced the chain mobility.^24^

**Figure 4:**
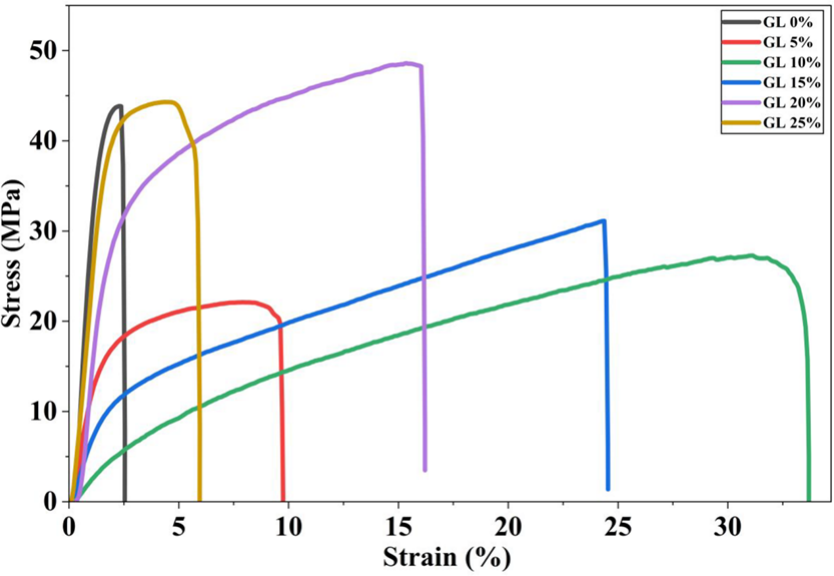
Tensile stress–strain curves of PVA/CS/GL films. GL addition significantly increases ductility, reaching a maximum at GL10 (>35% strain). GL15 maintains balanced strength and stretchability, while higher GL levels (GL20–GL25) increase stiffness but reduce elongation due to microstructural reinforcement.

An improvement in ductility was observed at 15 wt % GL (GL15), where the film reached an extended strain of ∼25% with a moderate tensile strength (∼30 MPa), indicating an optimal balance between flexibility and structural stability. At 20 wt % GL (GL20), the film exhibited the highest tensile strength among all GL-containing samples (>50 MPa) but with reduced elongation (∼16–17%), suggesting increased stiffness due to stronger hydrogen bond reinforcement from the zwitterionic functional groups of GL (NH_3_^+^/NH_2_ and −COO^–^). At the highest GL loading (25 wt %), the tensile curve showed further reduced elongation (∼6–7%) and moderate strength, indicating partial phase separation or excessive GL-induced rigidification. This suggests that beyond a critical concentration, GL may limit chain extensibility and weaken the overall deformability of the polymer network.^17^ The GL-dependent transition in flexibility and strength can be attributed to hydrogen bonding and molecular interactions among the −OH, −NH_3_^+^/NH_2_, and −COO^–^ groups. For TENG applications, tough yet flexible films are particularly advantageous because they require mechanically compliant materials capable of sustaining repeated deformation during operation.

### 3.4 Degradability Test

The biodegradation behavior of the PVA/CS/GL composite film was evaluated using a baseline hydrolytic immersion test by immersing a 10 × 10 mm specimen in phosphate-buffered saline (PBS; pH 7.4) at room temperature. At predetermined time intervals, the specimen was removed, gently rinsed with DI water to remove residual salts, and dried at 40 °C to a constant mass before weighing. The percentage weight loss was calculated as

$$\mathrm{Weight}\mathrm{loss}\left(\%\right)=\frac{W_{0}-W_{t}}{W_{0}}\times 100$$

where *W*_0_ is initial dry mass of the sample and *W*_*t*_ is the dry mass after immersion for time *t*.

The initial dry mass was 0.01106 g (day 1) and decreased progressively to 0.00908 g (day 2), 0.00822 g (day 3), 0.00709 g (day 4), 0.00510 g (day 5), 0.00397 g (day 6), and 0.00312 g (day 7) corresponding to an overall weight loss of ∼71.8% by day 7. Additional measurements at later time points showed further mass loss to 0.00255 g (day 9) and 0.00227 g (day 11), corresponding to ∼76.9% (day 9) and ∼79.5% (day 11). This rapid hydrolytic mass loss/disintegration is attributed to the hydrophilic and ionizable functional groups (NH_3_^+^/NH_2_ and −COO^–^) within the PVA/CS/GL network, which promote water uptake and dissolution/erosion processes.^15^

To provide quantitative degradation kinetics, the remaining mass fraction (Mt/M1, where M1 is the day 1 dry mass) and ln­(Mt/M1) were calculated from the measured masses (Figure 5). As shown in Figure 5, the film exhibited rapid hydrolytic degradation, reaching ∼71.8% weight loss by day 7 and ∼79.5% by day 11. An apparent hydrolytic degradation rate constant was estimated by fitting ln­(Mt/M1) versus immersion time using a pseudo-first-order model (ln­(Mt/M1) = −*kt*). The fit yields *k* ≈ 0.18 day^–1^ with *R* ^2^ ≈ 0.96 corresponding to an apparent half-life of $t_{1/2}=\ln\;2/k\approx 3.9$ days under PBS (pH 7.4, room temperature) conditions. The day-by-day photographic evolution is provided in the Supporting Information (Figure S5).

**Figure 5:**
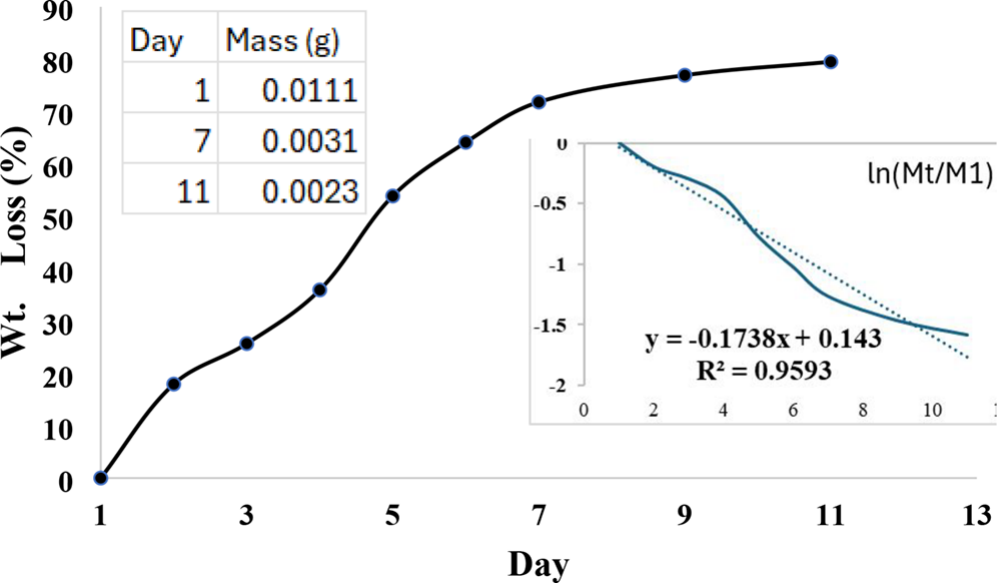
Hydrolytic degradation of the PVA/CS/GL composite film in PBS (pH 7.4) at room temperature, shown as weight loss (%) versus immersion time with pseudo-first-order kinetics of degradation. The inset table lists the measured remaining dry mass (g) at each time point. Representative photographs of the day-by-day degradation process are provided in Supporting Information (Figure S5).

PBS immersion provides a controlled hydrolytic baseline but does not capture the full complexity of soil, freshwater, or marine degradation pathways. Enzymatic degradation (e.g., lysozyme-containing media), identification of degradation products/intermediate species, and monitoring of electrical output during progressive degradation will be investigated in the future work to establish performance–lifetime coupling.

### 3.5 Electrical Performance of the EB-TENG

The electrical output behavior of the EB-TENG was evaluated in the vertical contact–separation mode, where the PVA/CS/GL film served as the tribo-positive layer and the PDMS film served as the tribo-negative layer (Figure 6a). The voltage and current signals generated during mechanical excitation were recorded in real time using a digital oscilloscope. Figure 6b illustrates the working mechanism of the EB-TENG device. Upon full contact between the two surfaces during the pressing stage, triboelectric charge transfer occurred at the interface, producing positive charges on the PVA/CS/GL film and negative charges on the PDMS layer (pressed state). As the shaker moves upward and the layers begin to separate, the electrostatic potential difference generated by the opposite surface charges drives electron flow through the external circuit, producing a current from the copper electrode on the PVA/CS/GL side toward the PDMS electrode (releasing state). When the layers reach maximum separation, electrostatic equilibrium is established, and no additional charge transfer occurs (released state). As the shaker moves downward and the gap decreases, the potential gradient reverses, causing electrons to flow in the opposite direction (pressing state). This cyclic approach–separation process produces a continuous alternating output. Device-to-device reproducibility was evaluated for the optimized GL20 formulation using *n* = 4 independently fabricated devices at 10 Hz, and the statistics (mean ± SD) are provided in Supporting Information (Table S3 and Figure S6).

**Figure 6:**
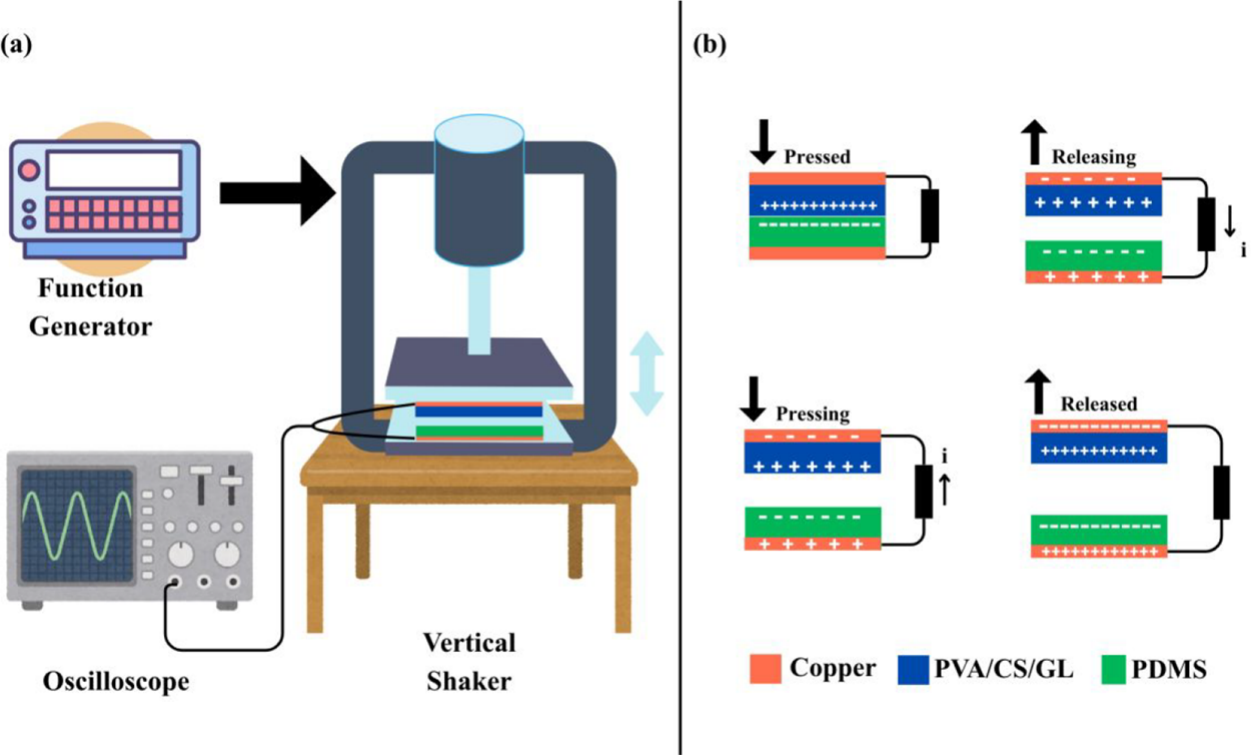
(a) Schematic illustration of the experimental setup and (b) working principle of the EB-TENG. (Attribution: illustration was created in Canva with icons by Paul J. from flaticon.com).

The frequency-dependent open-circuit voltage (*V*_oc_) characteristics of the optimized device are shown in Figure 7a. The TENG displayed a clear increase in the voltage amplitude as the operating frequency increased from 3 to 10 Hz, with the peak voltages increasing from only a few volts at low frequencies to over 70 V at higher frequencies. This improvement is attributed to the greater number of contact–separation cycles per unit time and enhanced impulsive force at higher frequencies, both of which facilitate a more rapid charge induction and stronger surface polarization.^25,26^ The well-defined and consistent waveform patterns observed at all frequencies confirmed the robust mechanical compliance and stable contact behavior during periodic operations.

**Figure 7:**
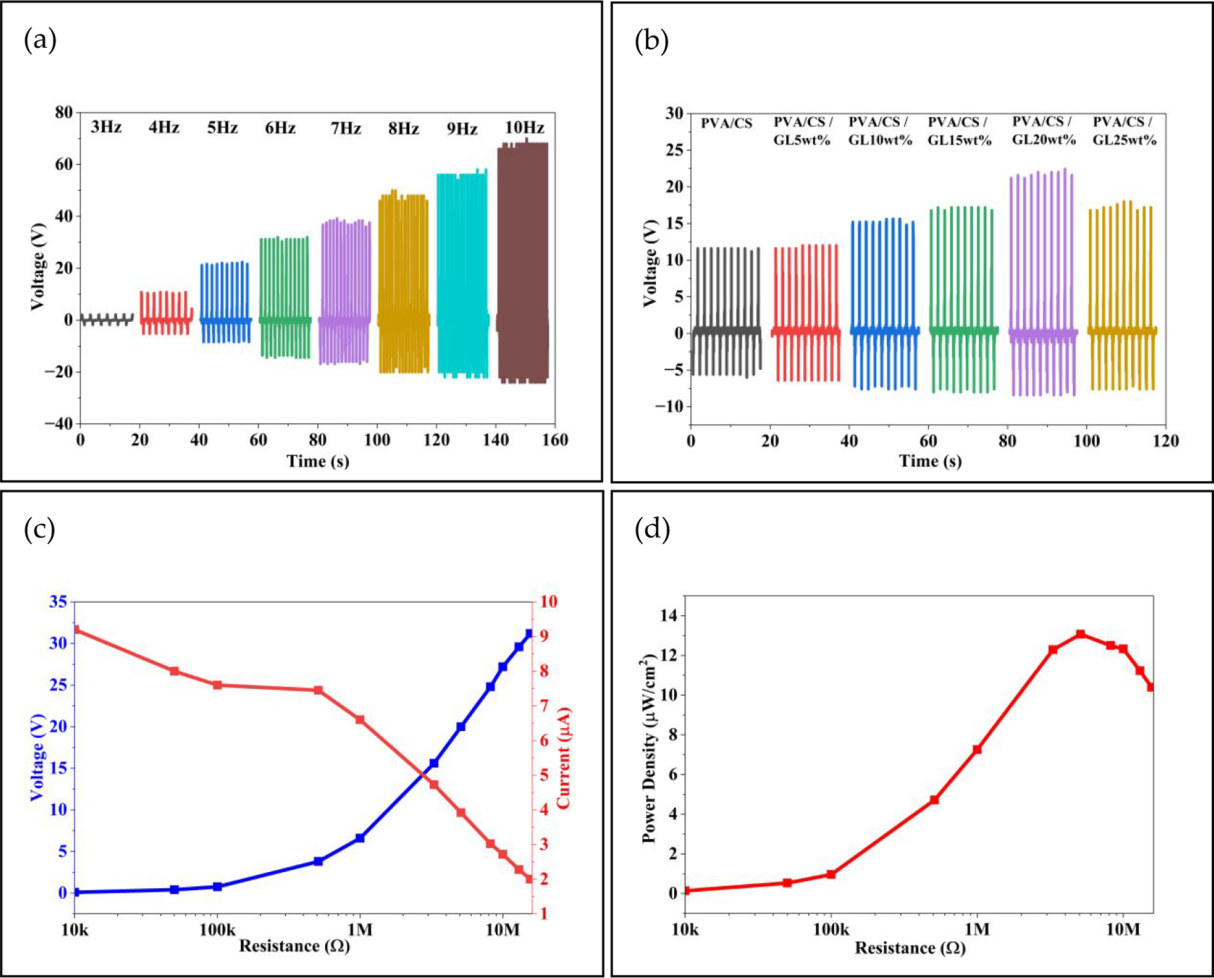
Electrical output characteristics of the EB-TENG: (a) *V*_oc_ at different excitation frequencies (3–10 Hz). (b) *V*_oc_ of films with varying GL concentrations (0–25 wt %) at 5 Hz. (c) Voltage and current under various external load resistances. (d) Power density as a function of load resistance. Representative current waveform at the matched load and long-term durability are provided in Supporting Information (Figure S7).

The effect of the GL concentration on the open-circuit voltage output is shown in Figure 7b. At a fixed excitation frequency of 5 Hz, the pristine PVA/CS film generated modest voltage signals, whereas the incorporation of GL progressively enhanced the output. The peak *V*_oc_ increased steadily with increasing GL content, reaching its highest value (∼36 V) at 20 wt % GL concentration. This enhancement is attributed to GL's zwitterionic functional groups (−NH_3_^+^ and −COO^–^), which increase the density of polar sites and can influence interfacial polarization and charge trapping/retention during contact–separation operation.^27^

Beyond the compositional effect, the GL-dependent electrical response is consistent with the structural and chemical changes observed in the composite films. FTIR confirms the presence of abundant polar functional groups (−OH, −NH_2_/–NH_3_^+^, and −COO^–^) and strong intermolecular interactions among PVA, CS, and GL, while SEM shows a roughened microtexture with GL-rich domains that can increase the effective contact area and provide additional charge-trapping sites at the interface. In a triboelectric contact–separation device, such polar-site enrichment and microstructural roughening can collectively enhance surface charge density and improve charge retention under repeated cycling, which aligns with the observed increase in the output upon GL incorporation. We note that this EB-TENG is designed and operated in a triboelectric mode; therefore, although β/γ GL polymorphs are often explored for stronger piezoelectricity in hybrid devices, we used commercially available α-GL to ensure reproducible solvent casting and systematic evaluation of GL-loading effects in a biodegradable matrix. Because α-GL is centrosymmetric, a significant intrinsic bulk piezoelectric contribution is not expected; thus, the GL-dependent enhancement observed here is discussed primarily in terms of triboelectric interfacial effects rather than a piezoelectric mechanism. Direct polymorph-resolved comparisons and quantitative surface potential/charge-trapping measurements (e.g., Kelvin probe force microscopy or electrostatic force microscopy) would provide mechanistic quantification of GL-induced charge retention and are identified as future work.

The voltage–current characteristics of the device under different external resistances (10 kΩ to 15.6 MΩ) are presented in Figure 7c. As the resistance increased, the output voltage increased progressively from a few volts at low resistances to over 30 V at high resistive loads. This steady increase is attributed to the reduced charge leakage and enhanced charge accumulation when the external impedance approaches the internal impedance of the TENG. In contrast, the current output decreased from 9.2 μA in the low-resistance region to 2 μA in the megohm range, reflecting the expected inverse relationship between the voltage and current. This opposing trend represents the classical load-dependent behavior of TENGs and confirms that the internal impedance of the device lies within the megaohm range, which is consistent with that of polymer-based triboelectric systems.

The power density variation as a function of the external load resistance is shown in Figure 7d. The device exhibited a low-power output at small resistances owing to insufficient voltage build-up, followed by a clear rise in the megohm region as the voltage and current reached an optimal balance. A maximum power density of 13.07 μW cm^–2^ was obtained at a load resistance of 5.1 MΩ, beyond which the power output gradually declined because of the reduced current at excessively high resistive loads. This bell-shaped load-matching profile is characteristic of TENG systems and demonstrates an optimal trade-off between voltage accumulation and charge-transfer efficiency. The current output at an optimal load resistance of 5.1 MΩ is shown in Figure S7a. The waveform exhibits sharp, periodic, and highly consistent current peaks associated with each contact–separation event. The peak current values reached up to 4 μA, indicating efficient charge flow during mechanical triggering and confirming the stable triboelectric response of the PVA/CS/GL composite device.

The long-term durability of the device is illustrated in Figure S7b. The *V*_oc_ output remained stable over more than 7500 continuous cycles at a tapping frequency of 5 Hz with no observable decline in the peak amplitude or waveform shape. The sustained voltage stability over an extended operation period demonstrates the mechanical robustness of the PVA/CS/GL composite film and confirms its suitability for long-term energy-harvesting and self-powered sensing applications.

To further contextualize the achieved output within biodegradable/biobased TENGs, we benchmarked our device against representative reports in the recent literature. As summarized in Table 1, biodegradable and biobased TENGs reported in the literature exhibit a wide range of electrical outputs depending on the material system and device design: a PVA/cellulose device delivers 60 V, 11.7 μA, and 410 μW cm^–2,^ ^28^ while a surface-roughened CS/glycerol TENG shows 38 V, 5.1 μA, and 11.22 μW cm^–2^.^11^ Incorporation of high-dielectric ceramic fillers can substantially boost output, as demonstrated by the CS–BaTiO_3_ composite with 111.4 V, 21.4 μA, and 756 μW cm^–2^.^29^ For GL-related architectures, a PVA/GL/PVA system reports 94 V and 2.3 μA with a power density of 6.96 μW cm^–2^ and degrades in PBS (37 °C, pH 7.4) within approximately 11 days.^18^ A bioresorbable BD-TENG based on PCL/PLGA/PVA/PHB-V reports 28 V, 0.6 μA, and 32.6 μW cm^–2^ with a rapid loss of function once water penetrates the device and the Mg electrode dissolves (≈24 h).^30^ Additional biodegradable polymer systems, such as CS/SF, achieve 77 V, 13 μA, and 22.4 μW cm^–2^ with soil burial degradation over 12 weeks,^31^ and a CS/glycerol device reports 50 V, 1.1 μA, and 27 μW cm^–2^ with complete soil burial degradation in ∼69 days.^7^ Cellulose-based TENGs have also been reported across a broad range of outputs (*V*_oc_ = 21–84 V; *I*_sc_ = 4.6–9.4 μA; power density = 1–25 μW cm^–2^), although degradation is not always reported.^32^

**Table 1:** Comparison of the Electrical Performance of PVA or CS-Based Biodegradable TENGs Reported in the Literature

**TENG materials**	***V*_oc_ (V)**	***I*_sc_ (μA)**	**power density** **(**μW cm^–2^ **)**	**degradation**	**ref**
PVA/cellulose	60	11.7	410	not reported	^28^
CS/glycerol	38	5.1	11.22	not reported	^11^
CS/BaTiO_3_	111.4	21.4	756	not reported	^29^
PVA/GL/PVA	94	2.3	6.96	PBS (37 °C, pH 7.4), ∼11 days	^18^
PCL/PLGA/PVA and PHB/V	28	0.6	32.6	PBS; Mg electrode dissolves within ∼24 h after water ingress	^30^
CS/silk fibroin	77	13	22.4	soil burial, 12 weeks	^31^
CS/glycerol	50	1.1	27	soil burial, complete in ∼69 days	^7^
cellulose/Ti3C2Tx	21, 35, 84	5.4, 9.4, 4.6	1, 3.5, 25	not reported	^32^
PVA/GL/CS	70	9	13.07	PBS; ∼72% mass loss within 1 week	this work

In this context, our PVA/GL/CS EB-TENG delivers 70 V, 9 μA, and 13.07 μW cm^–2^ while showing ∼72% mass loss within 1 week in PBS (baseline hydrolytic condition), supporting competitive performance among biodegradable polymer-based TENGs while maintaining clear degradability; nevertheless, direct numerical comparisons should be interpreted with caution because testing conditions (e.g., force, frequency, effective contact area, load resistance, and ambient humidity) are not standardized across studies. The maximum power density of 13.07 μW cm^–2^ suggests suitability for intermittent low-power operation when combined with rectification and energy storage; practical demonstration and application context are discussed in Section 3.6.

### 3.6 Applications

#### 3.6.1 Demonstrations

To validate the practical applicability of the developed EB-TENG, its capability to harvest biomechanical energy and power small electronic components was evaluated (Figure S8). As expected for a device operating in the vertical contact–separation mode, the EB-TENG generated an alternating current (AC) output during mechanical tapping. Because most portable electronics operate on direct current (DC), the AC signal was converted using a full-wave bridge rectifier to enable DC extraction for energy storage and load-driving applications (Figure 8a). Figure 8b shows successful lighting of an LED connected to the rectified output, demonstrating sufficient voltage build-up and charge transfer to overcome the diode threshold and function as a micropower source under low-frequency mechanical stimuli.

**Figure 8:**
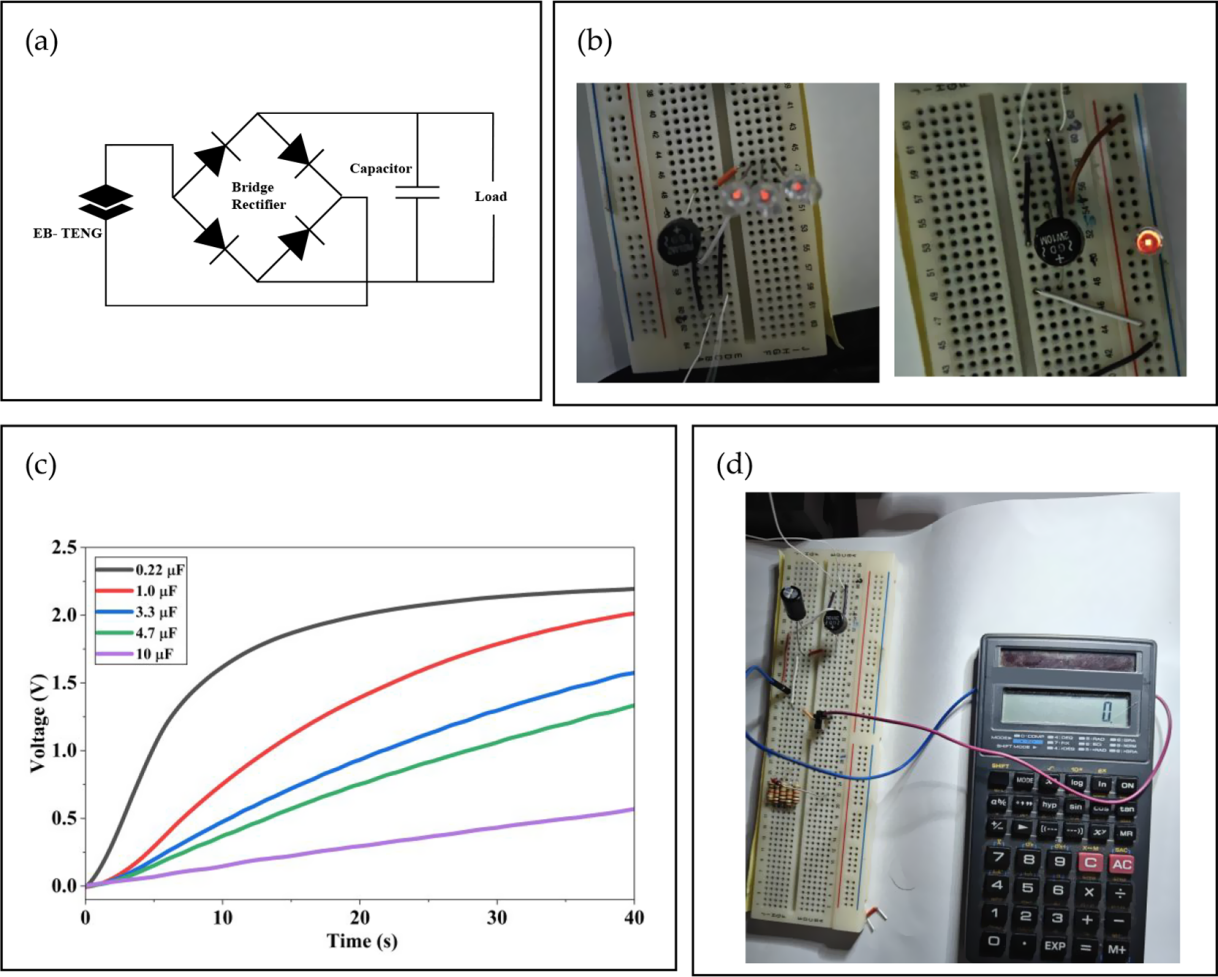
(a) Full-wave bridge rectifier used to convert the AC output of the EB-TENG to DC for charging and device operation. (b) LED lighting demonstration using the rectified output. (c) Capacitor-charging profiles (0.22–10 μF) driven by the rectified output under continuous tapping. (d) Demonstration of the EB-TENG powering a calculator using 470 μF storage capacitor.

The capacitor-charging behavior under continuous mechanical excitation is shown in Figure 8c. A clear capacitance-dependent trend was observed: the smallest capacitor (0.22 μF) charged most rapidly, reaching ∼2.3 V within 40 s, whereas larger capacitors (1.0, 3.3, 4.7, and 10 μF) exhibited progressively slower charging rates and lower voltages of ∼1.8, 1.3, 1.1, and 0.6 V, respectively, over the same duration. This inverse relationship arises because larger capacitors require a greater accumulated charge to reach a given voltage. The continuously increasing curves indicate persistent charge delivery during tapping and effective energy storage across capacitive loads.

To further demonstrate practical operation, a rectifier–capacitor module powered by the EB-TENG was used to operate an electronic calculator (Figure 8d). For the calculator demonstration, the rectified output was stored in a 470 μF capacitor prior to powering the load. The stored energy can be estimated by *E* = 1/2*CV* ^2^; for example, reaching 1.5 V corresponds to *E* ≈ 5.3 × 10^–4^ J (0.53 mJ), confirming storage-assisted, intermittent powering of a low-power electronic load. With continuous tapping, the stored energy provided sufficient voltage to activate the calculator display, highlighting the feasibility of powering low-power electronics using sporadic biomechanical inputs typical of hand-held or wearable scenarios. Overall, these demonstrations confirm that the EB-TENG can convert intermittent mechanical motion into usable electrical energy for small-scale devices and energy-storage elements.

#### 3.6.2 Practical Considerations (Performance, Lifetime, and Scalability)

The measured maximum power density (13.07 μW cm^–2^) places the EB-TENG within the lower range commonly reported as sufficient for intermittent operation of low-power wearable sensing systems (typically ∼10–50 μW, depending on duty cycle and power management).^4,33^ Accordingly, the most suitable use mode is pulse-type energy harvesting coupled with energy storage (capacitor/supercapacitor) and low-duty-cycle loads, rather than continuous high-power supply.^2,26^ In addition, the rapid mass loss observed in PBS suggests that the present hydrophilic, biodegradable formulation is best positioned for short-term/disposable green electronics, such as temporary wearable patches, single-use environmental sensors, and transient human–machine interfaces where end-of-life decomposition is desired.^6,30^ In contrast, long-term wearable deployment would require moisture-barrier encapsulation (or formulation tuning) to slow water uptake and degradation while maintaining output. Based on these considerations, we explicitly recommend the current device primarily for disposable/transient applications, and for wearable platforms only in a replaceable (cartridge-type) configuration or with appropriate encapsulation.

From a manufacturing perspective, the solvent-casting approach used here is compatible with scalable solution-coating routes, including doctor-blade and slot-die coating, which can be integrated into roll-to-roll processing for continuous production on flexible substrates.^34,35^ At larger scale, thickness uniformity and batch-to-batch reproducibility can be managed through controlled coating parameters (solution viscosity/solids content, coating gap, and coating speed) and controlled drying/conditioning (temperature and humidity), together with quality control (wet-film thickness monitoring and postdrying thickness checks).^35^ Spin coating is suitable for small-area uniform films and rapid screening, whereas slot-die and roll-to-roll coating are more appropriate for large-area, material-efficient manufacturing.^36–38^ Because the process uses aqueous/acetic acid-based solvents, closed-loop solvent recovery (e.g., capture/condensation of dryer exhaust) and recycling can be implemented to reduce solvent consumption and manufacturing waste.

A preliminary cost perspective is favorable because the tribo-positive layer consists of widely available commodity materials (PVA, CS, and GL) and does not require high-temperature processing, rare fillers, or sintering. A practical raw material cost estimate can be expressed as

Cost_film≈mass_PVA×Price_PVA+mass_CS×Price_CS+mass_GL×Price_GL(perdevice)

where mass is obtained from the film mass and formulation mass fractions. For a representative device area of 3 × 2 cm^2^, using the measured thickness range (∼0.042–0.056 mm) and a typical polymer film density (∼1.2 g cm^–3^), the total film mass is on the order of tens of milligrams, implying that the polymer-only cost per device is low and that electrodes, packaging/encapsulation, and assembly are likely to dominate the total device cost at the system level.

## 4 Conclusions

In this study, biodegradable and biocompatible PVA/CS/GL composite films were successfully fabricated using a simple solution-casting process and employed as triboelectric layers in TENG applications. By systematically varying the GL content from 0 to 25 wt % while maintaining a fixed PVA:CS ratio of 2:1, the structural, mechanical, and electrical contributions of GL to the composite matrix were determined. SEM and FTIR analyses confirmed that GL, in its zwitterionic form, enhances intermolecular hydrogen bonding, improves interchain compatibility, and induces GL-dependent surface texturing, which is favorable for triboelectric charge generation. Mechanical testing revealed that moderate GL incorporation (10–20 wt %) improved the film ductility and toughness through molecular bridging and controlled plasticization. Electrical measurements demonstrated that the composite containing 20 wt % GL achieved the highest output performance, producing an open-circuit voltage of approximately 70 V at 10 Hz and load-dependent current outputs reaching 9.2 μA at low resistance, with a ∼4 μA peak current at the optimal load of 5.1 MΩ. The maximum power density increased to 13.07 μW cm^–2^ at the 5.1 MΩ load, confirming the favorable load-matching behavior. The device exhibited highly stable output performance over more than 7500 continuous cycles, maintaining a consistent voltage amplitude and waveform shape, which highlights its excellent mechanical robustness and long-term operational reliability. The degradability of the film was further confirmed through hydrolytic testing, which showed up to 72% mass loss within 1 week. Additionally, the rectified output effectively powered LEDs and demonstrated efficient energy storage by charging capacitors from 0.22 to 10 μF, with the 0.22 μF capacitor reaching ∼2.3 V within 40 s. These results confirm the practical feasibility of the PVA/CS/GL-based EB-TENG for powering small electronic devices. Overall, this study presents a green, low-toxic, and scalable strategy for designing biodegradable triboelectric materials with tunable performance. The synergistic integration of PVA, CS, and GL provides a promising platform for wearable electronics, disposable sensors, and environmentally sustainable self-powered systems.

## Supplementary Material



## Data Availability

Data set is available upon request from the authors.

## Notes

EB-TENG preparation and assembly photographs; high-magnification SEM micrographs of PVA/CS/GL composite films; EDS spectra of GL0 and GL20 films; tensile testing setup photograph; PBS degradation photographs; device-to-device reproducibility of the EB-TENG output; matched-load current waveform and long-term durability over 7500 cycles; LED and calculator powering demonstrations; film thickness and within-film thickness uniformity; EDS-derived elemental composition; and device-to-device reproducibility summary for the optimized EB-TENG (PDF)
