# Supplementary material for: Poly(vinyl alcohol)/Chitosan/Glycine Composite-Based Eco-Friendly Biodegradable Triboelectric Nanogenerators
Source: ACS Omega. 2026 May 16;11(21):30675–85. doi: 10.1021/acsomega.5c12532 (PMC13234806; doi:10.1021/acsomega.5c12532)
Supplement: Supplementary file 1 [file ao5c12532_si_001.pdf]

Supporting Information

Polyvinyl alcohol/Chitosan/Glycine Composite -based Ecofriendly Biodegradable Triboelectric Nanogenerator

Tulsi Paudel[1], Cheng-Tang Pan[1234]*, Cheng-Yi Chen[5], Yow-Ling Shiue[46], Muhammad Sadiq Rahim[6]

[1] Department of Mechanical and Electro-Mechanical Engineering, National Sun Yat-sen University, Kaohsiung 804201, Taiwan (R.O.C)
[2] National Center for Instrumentation Research, National Institutes of Applied Research, Hsinchu City 300092, Taiwan (R.O.C)
[3] Institute of Advanced Semiconductor Packaging and Testing, College of Semiconductor and Advanced Technology Research, National Sun Yat-sen University, Kaohsiung 804201, Taiwan (R.O.C)
[4] Institute of Precision Medicine, National Sun Yat-sen University, Kaohsiung 804201, Taiwan (R.O.C)
[5] Department of Electrical Engineering, Cheng Shiu University, Kaohsiung 833301, Taiwan (R.O.C)
[6] Institute of Biomedical Sciences, National Sun Yat-sen University, Kaohsiung 804201, Taiwan (R.O.C)

***** Correspondence: pan@mem.nsysu.edu.tw (C.T. Pan)

Contents

Figures:

Figure S1. Preparation and assembly steps of the EB-TENG.

Figure S2. High-magnification SEM micrographs of composite films.

Figure S3. EDS spectra of the composite films.

Figure S4. Photograph of the tensile testing setup for the composite film.

Figure S5. Photographic time series of PVA/CS/GL film degradation in PBS.

Figure S6. Device-to-device reproducibility of the EB-TENG output.

Figure S7. Current waveform and durability test of the EB-TENG.

Figure S8. Demonstration of the EB-TENG powering small electronic loads.

Tables:

Table S1. Thickness and within-film thickness uniformity of composite films.

Table S2. EDS-derived elemental composition composite films.

Table S3. Summary of device-to-device reproducibility for the optimized EB-TENG.

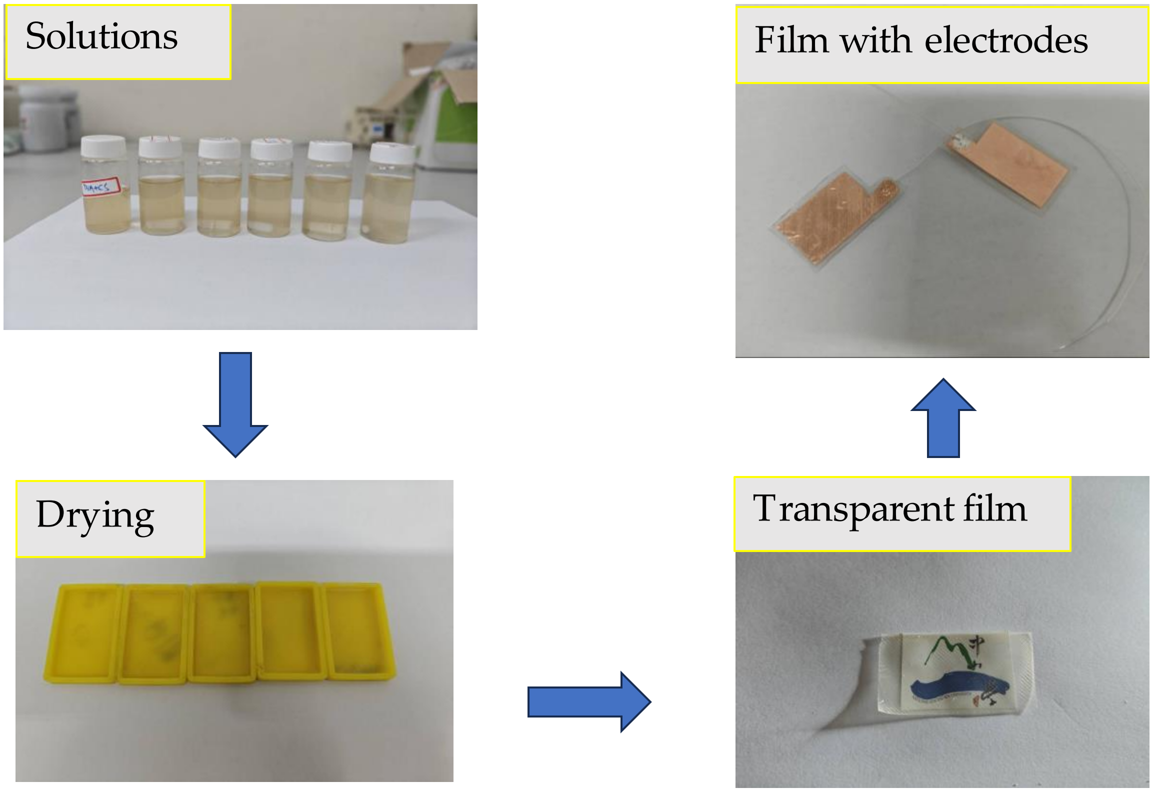

Figure S1. Preparation and assembly steps of the EB-TENG. Photographs showing solution preparation, drying after solvent casting, the obtained transparent composite film, and the film integrated with Cu electrodes.

Table S1. Thickness and within-film thickness uniformity of composite films. Thickness was measured at five locations (center and four corners) using a digital micrometer after drying; values are reported as mean ± SD (n = 5 points per film), and CV (%) indicates within-film thickness variation.

Film	t1(mm) (Center)	t2 (mm) (Corner1)	t3 (mm) (Corner2)	t4 (mm) (Corner3)	t5(mm) (Corner4)	Mean ± SD (mm)	CV (%)
Film 1	0.06	0.05	0.04	0.06	0.07	0.056 ±0.011	20.36
Film 2	0.05	0.04	0.04	0.03	0.05	0.042 ±0.008	19.92
Film 3	0.05	0.06	0.06	0.05	0.04	0.052±0.008	16.08

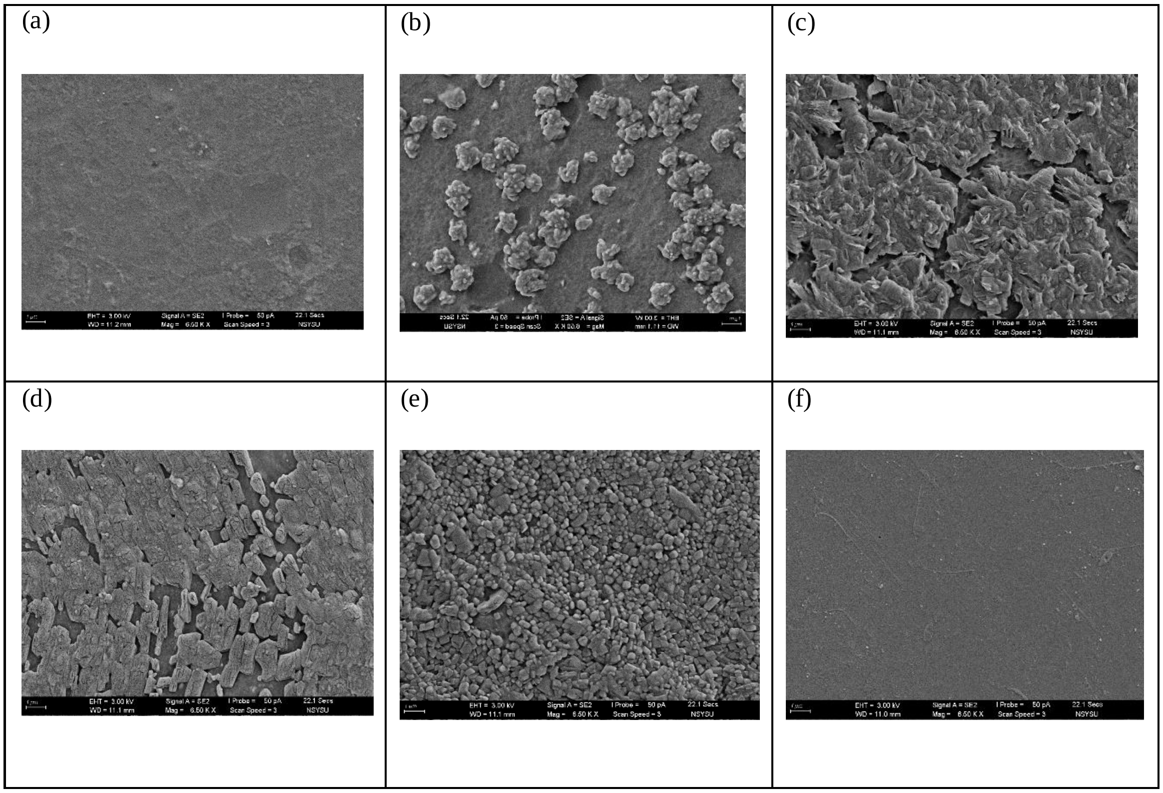

Figure S2. High-magnification SEM micrographs (1 μm scale) of PVA/CS/GL composite films as a function of glycine loading. (a) GL0, (b) GL5, (c) GL10, (d) GL15, (e) GL20, and (f) GL25. The images show glycine-dependent evolution of surface microtexture, including the emergence and growth of granular/crystallite-like domains at intermediate loadings. Scale bar: 1 μm.

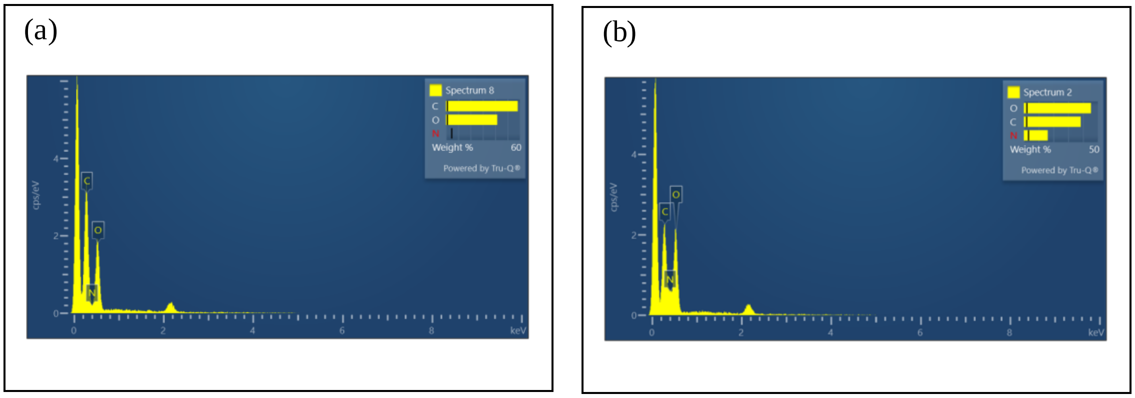

Figure S3. EDS spectra of the composite films: (a) PVA/CS film without glycine (GL0) and (b) PVA/CS/GL film with glycine (GL20). Nitrogen is not detected in GL0, while a clear nitrogen signal appears in GL20, consistent with glycine incorporation.

Table S2. EDS-derived elemental composition of PVA/CS (GL0) and PVA/CS/GL (GL20) films.

Element	GL0 wt.%	GL0 at. %	GL20 wt.%	GL20 at. %
C	58.29	65.06	38.43	44.49
N	0	0	16.18	16.06
O	41.71	34.94	45.39	39.45
Total	100	100	100	100

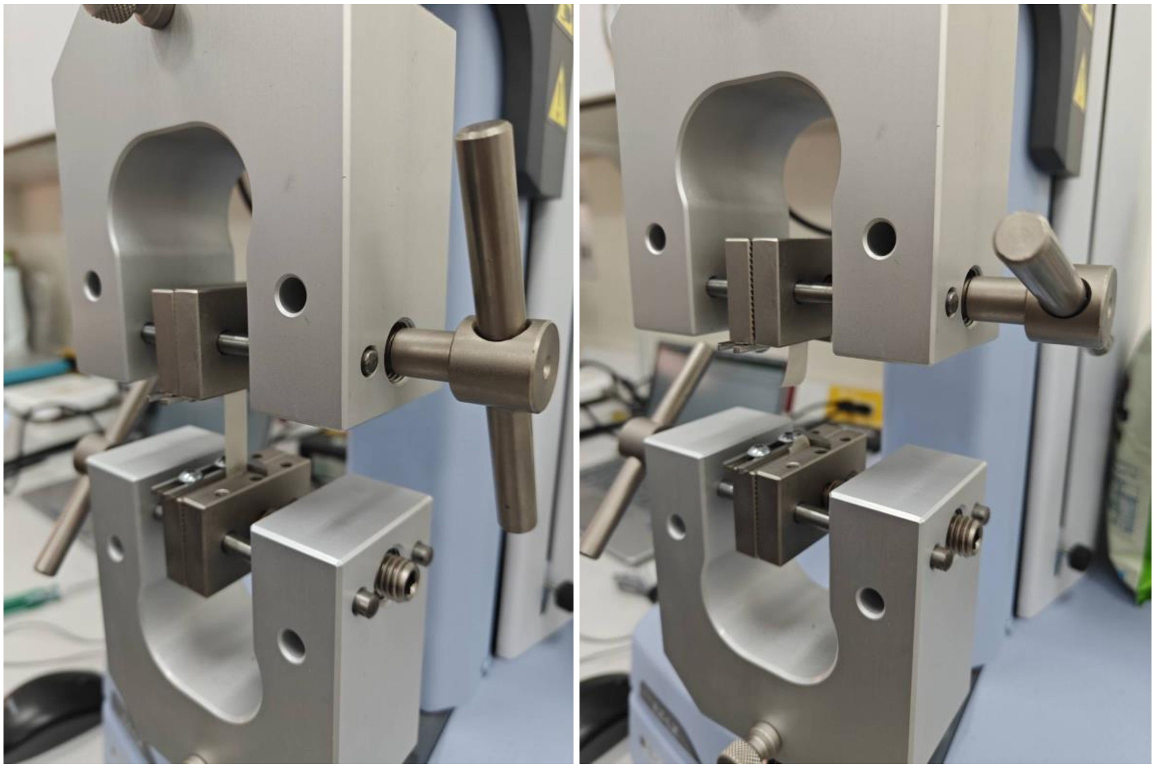

Figure S4. Photograph of the tensile testing setup for the composite film. Representative image of the PVA/CS/GL film mounted in the universal testing machine grips during uniaxial tensile testing (Shimadzu EZ-LX).

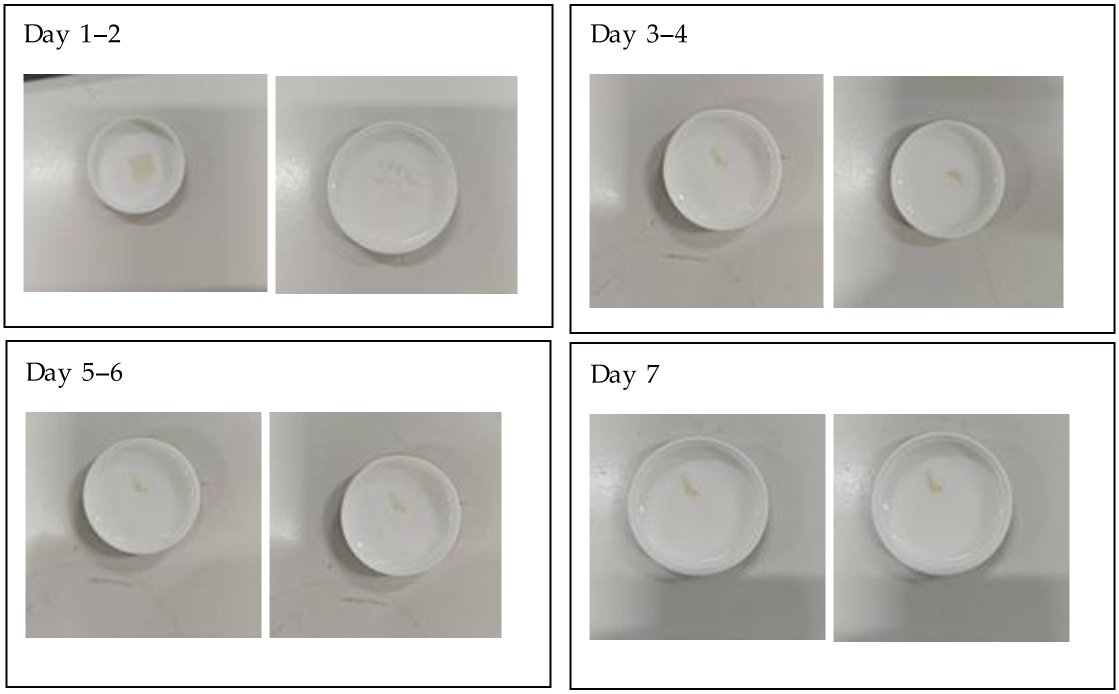

Figure S5. Photographic time series of PVA/CS/GL film degradation in PBS (pH 7.4). Images captured over 7 days at room temperature showing progressive disintegration of the composite film.

Statistical analysis of TENG electrical performance

Four identically fabricated TENG devices were characterized under identical loading conditions (Frequency 10Hz, force 10N) Key output parameters are summarized in the supplementary table S3 and Figure S7.

Table S3. Summary of device-to-device reproducibility for the optimized EB-TENG (GL20)

Parameter	Device 1	Device 2	Device 3	Device 4	Mean ± SD
Voc(V)	70	68.4	67	69	69.6± 1.25
Isc(μA)	9.2	8.4	8	8.68	8.57± 0.50
Power Density (μW cm^{-2})	13.07	12.40	11.16	12.38	12.25± 0.79

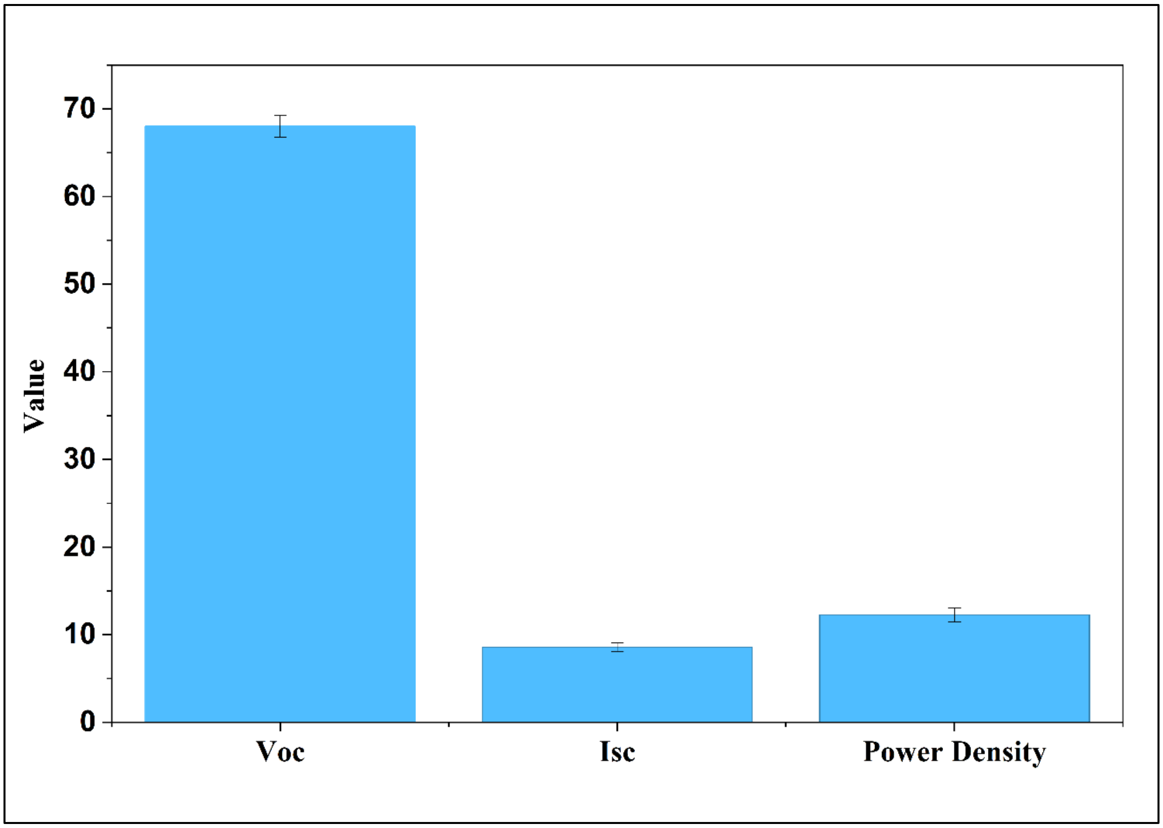

Figure S6. Device-to-device reproducibility of the EB-TENG output (optimized GL20 formulation).

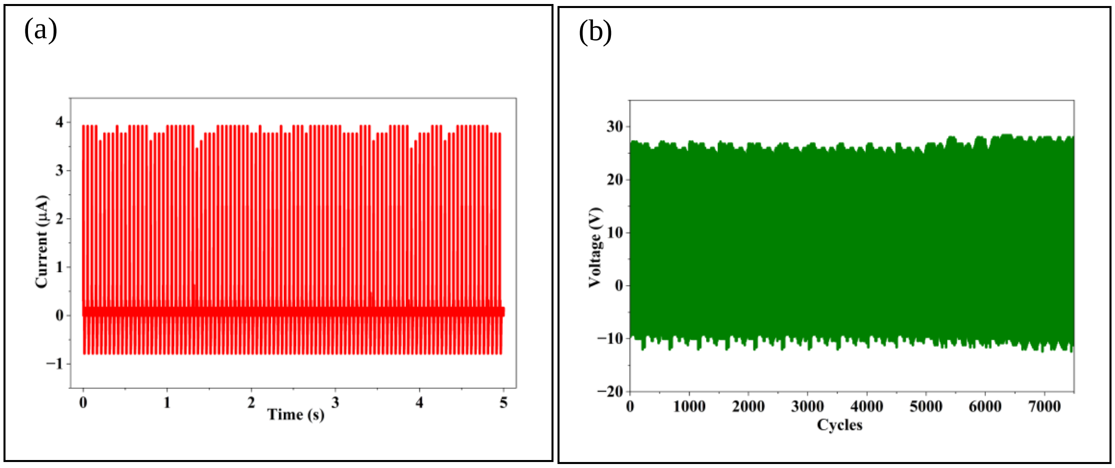

Figure S7. (a) Current waveform measured at the matched load resistance (5.1 MΩ). (b) Long-term durability of the EB-TENG over 7500 cycles at 5 Hz.

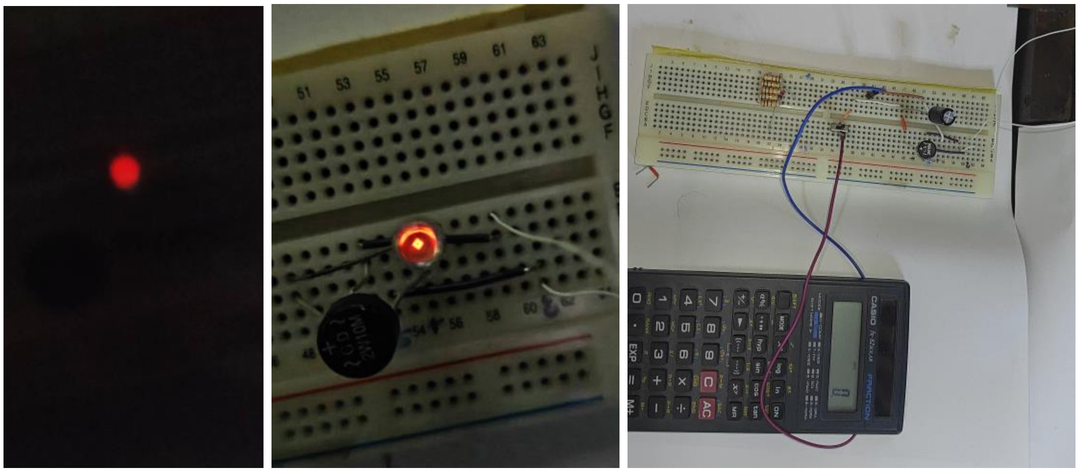

Figure S8. Demonstration of the EB-TENG powering small electronic loads. Photographs showing LED illumination and operation of an electronic calculator using the rectified output of the EB-TENG under manual tapping.
